# Vacancy control in acene blends links exothermic singlet fission to coherence

**DOI:** 10.1038/s41467-021-25395-9

**Published:** 2021-08-26

**Authors:** Clemens Zeiser, Chad Cruz, David R. Reichman, Michael Seitz, Jan Hagenlocher, Eric L. Chronister, Christopher J. Bardeen, Roel Tempelaar, Katharina Broch

**Affiliations:** 1 Institute of Applied Physics, University of Tübingen, Tübingen, Germany; 2 Department of Chemistry, University of California at Riverside, Riverside, CA USA; 3 Department of Chemistry, Columbia University, New York, NY USA; 4 Institute of Inorganic Chemistry, University of Tübingen, Tübingen, Germany; 5 University of Nevada, Las Vegas, 4505S. Maryland Pkwy, Las Vegas, NV USA; 6 Department of Chemistry, Northwestern University, Evanston, IL USA

**Keywords:** Excited states, Fluorescence spectroscopy

## Abstract

The fission of singlet excitons into triplet pairs in organic materials holds great technological promise, but the rational application of this phenomenon is hampered by a lack of understanding of its complex photophysics. Here, we use the controlled introduction of vacancies by means of spacer molecules in tetracene and pentacene thin films as a tuning parameter complementing experimental observables to identify the operating principles of different singlet fission pathways. Time-resolved spectroscopic measurements in combination with microscopic modelling enables us to demonstrate distinct scenarios, resulting from different singlet-to-triplet pair energy alignments. For pentacene, where fission is exothermic, coherent mixing between the photoexcited singlet and triplet-pair states is promoted by vibronic resonances, which drives the fission process with little sensitivity to the vacancy concentration. Such vibronic resonances do not occur for endothermic materials such as tetracene, for which we find fission to be fully incoherent; a process that is shown to slow down with increasing vacancy concentration.

## Abstract (web summary)

A complete understanding of singlet fission (SF) in molecular materials will enable the design of optimised optoelectronic devices. Here, the authors use vacancy control in acene-based blends to link coherent and incoherent SF pathways to energetics.

## Introduction

Singlet fission (SF), the spontaneous decay of a photoexcited singlet exciton into two triplet excitons in organic molecular materials, poses a fundamental conundrum as well as a promising avenue for the optimisation of photovoltaics and spintronics^1–4^. Great strides have been made in improving our mechanistic understanding of the SF process. A spin-correlated pair of triplets has been recognised as an important SF intermediate^4–6^, by rendering the process spin allowed, as well as charge-transfer states that directly mix with the singlet exciton, causing the nonadiabatic coupling to the triplet-pair state to effectively entail the transfer of a single electron instead of a concerted two-electron transfer^7–13^. Among other factors that have been deemed important are an approximate energetic resonance between the singlet and triplet-pair states, as well as discrete vibrational modes, in particular those resonant with the energy mismatch between the singlet and triplet-pair states^9,14–23^.

**Fig. 1:**
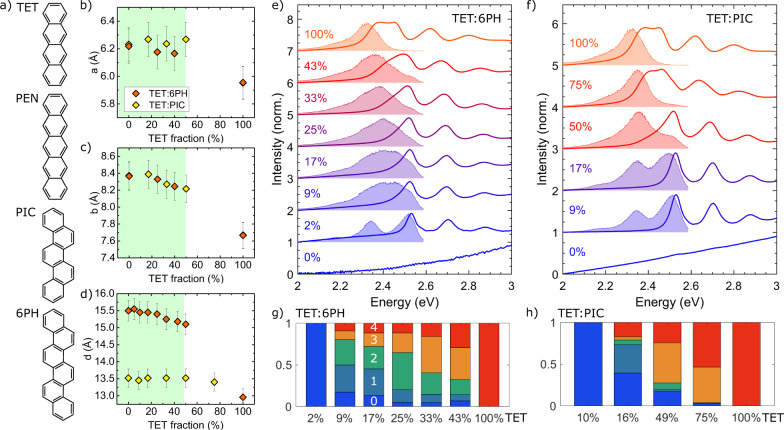
Structural and optical properties of TET blends. **a** Chemical structures of TET, PEN, PIC and 6PH. **b**–**d** In-plane unit cell parameters *a* and *b* and out-of-plane lattice spacing *d* for TET blends with varying concentrations of spacer molecules. The areas shaded in light green denote the range of TET fractions relevant for this study. The legend in **b** applies to **b**–**d**. The error bars have been determined as 2% of the experimental value. **e**, **f** Absorption spectra (solid lines) and photoluminescence (PL) spectra (shaded areas), offset for clarity, for **e** TET:6PH blends and **f** TET:PIC blends. **g**, **h** Decomposition of the PL spectra with differing TET fractions into five contributions, depending on the number of TET neighbours for a given TET molecule (numbers given in the bars; see also Figs. S6-S8). The monomer contribution corresponds to 0 TET neighbours while the bulk contribution corresponds to 4 TET neighbours. For details, see text.

These efforts notwithstanding, a systematic understanding of SF remains lacking, in particular how the combination of all known relevant factors synergistically steers SF under *both* exothermic and endothermic conditions. Among exothermic SF materials, crystalline pentacene (PEN, C_22_H_14_, see Fig. 1a) has been widely considered as a successful prototype where SF is found to be efficient and prompt^24,25^. However, a variety of endothermic materials have been shown to reach a similar SF efficiency while not suffering from a loss of energy in the singlet-to-triplet conversion event. The prototypical endothermic SF material is crystalline tetracene (TET, C_18_H_12_, see Fig. 1a), which among SF materials exhibits triplet excitons that are best suited for applications in conjunction with silicon-based photovoltaics^25–27^. This offers the prospect of utilising SF under optimised conditions, but the precise manner in which different energetic alignments (exothermic vs. endothermic) affect SF kinetics and yields has yet to be determined. While in the incoherent limit the energetic alignment defines the activation energy in a standard Arrhenius rate process, several studies have proposed that coherent mixing between the singlet and triplet-pair states can play an important role in SF^11,28–37^. The involvement of coherent mixing is challenging to probe experimentally and is commonly inferred when SF is unusually fast and/or shows a lack of temperature dependence^11,30–33,38^. However, such behaviour can potentially reflect other processes^18,39^, and complementary approaches to substantiate the importance of coherence in SF are highly desirable.

Here, we show a remarkable difference in the role of coherence between exothermic and endothermic SF in mixed thin films of PEN and TET codeposited with high-band gap organic compounds. These compounds act as spacer molecules: noninteracting replacements of PEN or TET, which effectively form vacancies, the concentration of which enables us to accurately control the average number of neighbours for each SF molecule, but which leave the crystal structure practically unchanged. As such, this approach differs from approaches based on the incorporation of SF chromophores in amorphous host materials^40^, which allow to study SF under pronounced variations in intermolecular distances instead.

We utilise the present approach as a complementary means to interrogate the coherent behaviour of PEN- and TET-based blends using time-resolved spectroscopy, an approach that is substantiated by accompanying computational modelling that reproduces the key experimental observations and quantifies the degree of coherent mixing in microscopic detail. The two SF molecules considered in our study allow us to compare the SF process under exothermic and endothermic conditions. Previously, some of us observed the rate of exothermic SF in PEN-based blends to be robust against variations in the concentration of spacer molecules^41^. In the present work, the SF rate of endothermic TET-based blends is found to monotonically decrease upon adding spacer molecules, scaling with the average number of available neighbouring TET molecules. We demonstrate numerically that the completely different behaviours of PEN and TET result from the presence and absence of coherent SF, respectively, which is rationalised by the possibility and impossibility of energetic resonances between the relevant singlet and triplet-pair states generated by vibrational modes under exothermic and endothermic conditions.

## Results

### Structural and optical characterisation of the blends

Similar to PEN, neat TET deposited on weakly interacting substrates such as quartz glass grows in a herringbone arrangement with the long molecular axis almost perpendicular to the substrate plane^42^, such that each molecule has four neighbours in the plane parallel to the substrate where the intermolecular interactions are the strongest. The noninteracting spacer molecules used as vacancies in our TET blends were [6]-phenacene (6PH, C_26_H_16_) and picene (PIC, C_22_H_14_); see Fig. 1a. We performed a combined structural and optical characterisation of the blends in order to confirm statistical intermixing of TET and spacer molecules and to exclude cocrystal formation or phase separation.

The in-plane unit cell parameters *a* and *b*, obtained by X-ray diffraction, are shown in Fig. 1b,c and in the Supporting Information. With a decreasing concentration of TET molecules, both parameters show a small but continuous increase, indicating statistical mixing with a uniform random occupation of lattice sites by either TET or spacer molecules. For TET fractions exceeding 50%, we observe limited intermixing^43,44^, leading to a phase separation between neat TET domains and mixed domains. For this reason, we focused our experimental analysis on blends with TET fractions below 50%. Within this range, the variations in unit cell parameters are very small, suggesting that the electronic couplings between TET molecules are comparable across the relevant range of TET fractions.

For comparison, the in-plane unit cell parameters of the complementary system PEN mixed with PIC and diindenoperylene (DIP)^41,43^ are shown in the Supporting Information, exhibiting a behaviour similar to the TET-based blends, although variations with spacer concentration are slightly larger. The variation of the out-of-plane lattice spacing *d* with the TET or PEN fraction is shown in Fig. 1d and the Supporting Information, respectively, where it should be noted that electronic couplings along this direction are negligible owing to the overall larger molecular distances and minimal wavefunction overlap.

More information on the electronic properties of the blends is provided by linear-absorption spectroscopy. Shown in Fig. 1e, and 1f are absorption spectra taken for the TET:6PH and TET:PIC blends in the 2.0 eV - 3.0 eV range, which can be unambiguously assigned to the electronic transitions of TET molecules since 6PH and PIC have band gaps of 3.1 eV and 3.2 eV, respectively. With decreasing TET fraction, the shape of the absorption spectra evolves from the neat TET thin-film spectrum to the monomer spectrum, with distinct peaks that are blueshifted compared with the position in the neat-film spectrum by about 100 meV at a TET fraction of 2%, indicating that the majority of TET molecules have become completely isolated. Besides the blueshift due to changes in the polarizability of the molecular environment, the main spectral change with decreasing TET fraction is manifested in the Davydov components, which are the two lowest-lying absorption peaks located at 2.38 eV and 2.46 eV for neat TET. The energetic separation between these components, the Davydov splitting (DS), results from electronic interactions between the two translationally inequivalent molecules in the unit cell. In going from 50% to 5% TET fraction, the DS is seen to decrease monotonically from about 56 meV to 32 meV (see Supporting Information), indicating a decrease in the admixture of charge-transfer states in the lowest-lying electronic transition^7,12,41^. We note that the changes in unit cell parameters observed in our TET blends are a factor of 2 smaller than those observed previously for tetracene polymorphs^45^, including a fairly constant unit cell angle γ contrasting the 3° angular difference observed for the latter^42^. As such, structure-induced variations in the electronic couplings are expected to be markedly less pronounced in our TET blends than in the noted polymorphs^45,46^.

The steady-state photoluminescence (PL) spectra taken for the TET blends, shown in Fig. 1e, and 1f, mirror the continuous change of the spectral shape from neat TET to that of isolated monomers. Due to the statistical distribution of spacer molecules within the film, TET molecules in the blend have a varying number of other TET molecules as in-plane neighbours ranging from 4 (the bulk limit) down to 0 (the monomer limit), with the relative contribution of these configurations changing with TET fraction. These variations are borne out in Fig. 1g, and 1h, where the PL spectrum is deconvolved into spectral contributions from such configurations. The difference between the results of the decomposition for TET:6PH and TET:PIC blends is attributable to differences in the steric compatibility of the molecules and in the symmetry of the unit cell (see Supporting Information for details). Overall, the distribution of configurations resulting from this PL deconvolution deviates slightly from the binomial distribution expected for randomly intermixed TET and spacer molecules. The observed deviations are consistent with PL being preceded by exciton hopping to low-energy sites with an increased number of neighbouring TET molecules, which lower the energy locally through intermolecular interactions, as proposed before^41,47^.

### Time-resolved spectroscopy and determination of singlet fission rates

**Fig. 2:**
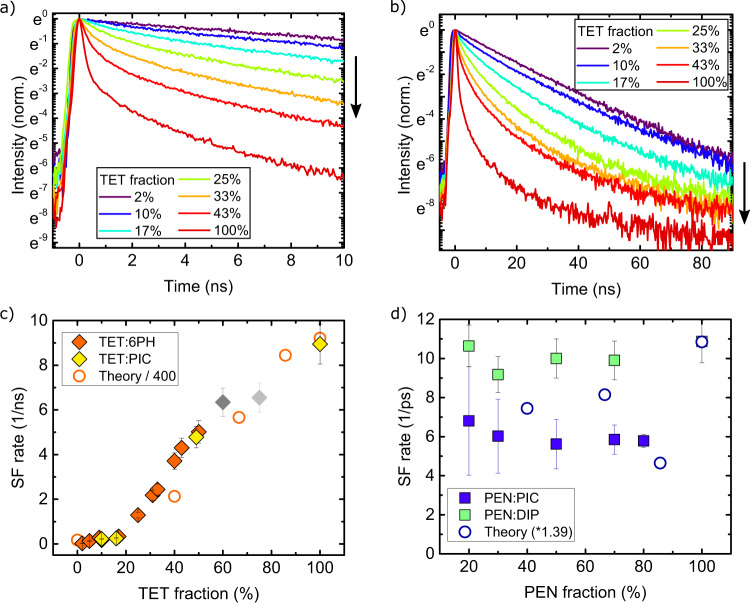
Time-resolved photoluminescence (TRPL) measurements and comparison of measured and calculated SF rates. **a** TRPL intensity of TET:6PH blends with different TET fractions in a 10-ns window. **b** Equivalent to **a** but in a 90-ns window. The arrows in **a** and **b** denote the direction of increasing TET fraction. **c**, **d** Comparison of experimentally (filled symbols) and theoretically (open circles) determined SF rate constants for TET- and PEN-based^41^ blends with varying concentrations of spacer molecules or the average number of nearest neighbours (NN). The experimental SF rates of TET:6PH and TET:PIC blends in **c** have been obtained by subtracting the monomeric decay rate (determined by fitting the 2% TET blend) from the decay rate of the prompt fluorescence, as explained in the text. The grey data points correspond to TET fractions where a phase separation of TET crystallites sets in (dark grey: TET:6PH, light grey: TET:PIC). The experimental data of PEN blends in **d** are adapted from Ref. ^41^. Error bars denote the 95% confidence interval of the fit.

In order to determine the SF dynamics in the TET blends, we performed time-resolved photoluminescence (TRPL) spectroscopy. The TRPL intensity shown in Fig. 2a,b is indicative of the time-dependent photoexcited singlet state population, and features a prompt, subnanosecond decay followed by a low-amplitude, longer-lived tail. These fast and slow components have in previous reports on neat TET thin films been assigned to SF and delayed fluorescence from triplet–triplet annihilation, respectively^25,48^. At high TET concentrations, the latter attribution is confirmed by its magnetic field dependence. For decreased TET concentrations, however, the slow component does not change with the applied magnetic field (see Supporting Information), indicating that it is not due to enhanced triplet–triplet annihilation^49^, and we instead assign it to singlet state emission from fully isolated TET monomers within the blends. Importantly, the fast component is seen to dramatically slow down with decreasing TET fraction. In the following, we focus on this component, as it reflects the upper limit of the concentration-dependent SF rate.

Based on the shape of the PL spectrum and the monoexponential decay of the PL intensity, we assume that the dynamics of the blend with the lowest TET fraction (2%) is dominated by isolated TET molecules, and that we can extract the concentration-independent monomeric decay-rate constant (*k*_monomer_) from a fit of these data^50^. For the blends with higher TET fractions (*f*), the total rate constant of the prompt decay is given by *k*_SF_(*f*) + *k*_monomer_ where the fraction-dependent contribution *k*_SF_(*f*) can be assigned to SF and is obtained by fitting a monoexponential decay function to the prompt decay of the TRPL measurements, see Supporting Information for details.

The fraction-dependent SF rate constants, *k*_SF_(*f*), are shown in Fig. 2c. Starting from a TET fraction of 50%, we observe a steep drop of the SF rate with decreasing TET fraction from 7 ns^−1^ for neat TET films to 0.3 ns^−1^ for a TET fraction of 10%. Both blends of TET with 6PH or PIC exhibit identical behaviour, from which we conclude that the reduced SF rate is a general result of vacancies introduced by spacer molecules, and not due to specific interactions of these molecules with TET. Reproduced in Fig. 2d are data for PEN mixed with PIC and DIP, which exhibit an invariance of the SF rate with PEN concentration, as previously discussed^41^ and where limited intermixing can be excluded for the latter^41^. The constant offset found between the two PEN-based blends is likely attributable to the difference in the polarizability of the local molecular environment experienced by a given PEN molecule in the presence of either DIP or PIC, which modulates the resonance between the singlet and triplet-pair states.

## Discussion

Previously, the observed insensitivity of the SF rate of the PEN-based blends was rationalised by fast hopping of singlet excitons to low-energy hot spots where SF proceeds effectively^41,47^. In this regard, the strong concentration dependence of the SF rate found for the TET-based blends is surprising, as the comparatively lower SF rate constants for TET in combination with high hopping mobilities^51^ should enable even more excitons to reach such hot spots. The potential contribution of spacer molecules to the exciton mobility (through superexchange) is expected to only further increase this contrast as their band gaps lie closer to TET than to PEN. Likewise, given that changes in the unit cell parameters are smaller for TET than for PEN, where the SF rate does not change, we expect modulations of the interaction strength between TET molecules resulting from structural changes to provide only a minor contribution to this concentration-dependent SF rate. We thus find an unexpected contrast in vacancy-dependent SF between the exothermic and endothermic blends, which potentially holds information on the mechanistic principles steering this process.

To obtain a microscopic insight into the SF dynamics for the TET-based and PEN-based blends, we performed calculations employing a theoretical model based on previous studies on neat TET^7^ and PEN^9,18^ crystals, here extended to include vacancies. In short, the acenes are represented using a limited selection of electronic states, including the (singlet) ground and first singlet excited states, ionic (electron) and cationic (hole) states, and the first triplet state, each of which is linearly coupled to a single intramolecular vibration (*ω*_0_). The dynamics under the corresponding Holstein Hamiltonian is calculated by Markovian Redfield theory in the secular approximation, assuming a Debye spectral density for the vibrational modes other than *ω*_0_ (for details, see Supporting Information and Refs. ^9,18^). The vacancies are considered as empty sites that are distributed over the crystal lattice, such that the average number of nearest neighbours for a given molecule equals the statistically expected value for blends with different TET and PEN fractions. This approach obviously does not involve phase separations between mixed and neat TET domains, so that the entire range of concentrations is at our disposal to study the SF process in our calculations. The unit cell parameters and intermolecular couplings were kept constant, allowing us to focus entirely on the effect of vacancies on the SF rates, although future efforts to further elucidate the potential effect of unit cell parameter variations as discussed in Refs. ^12,41^ would be interesting.

**Fig. 3:**
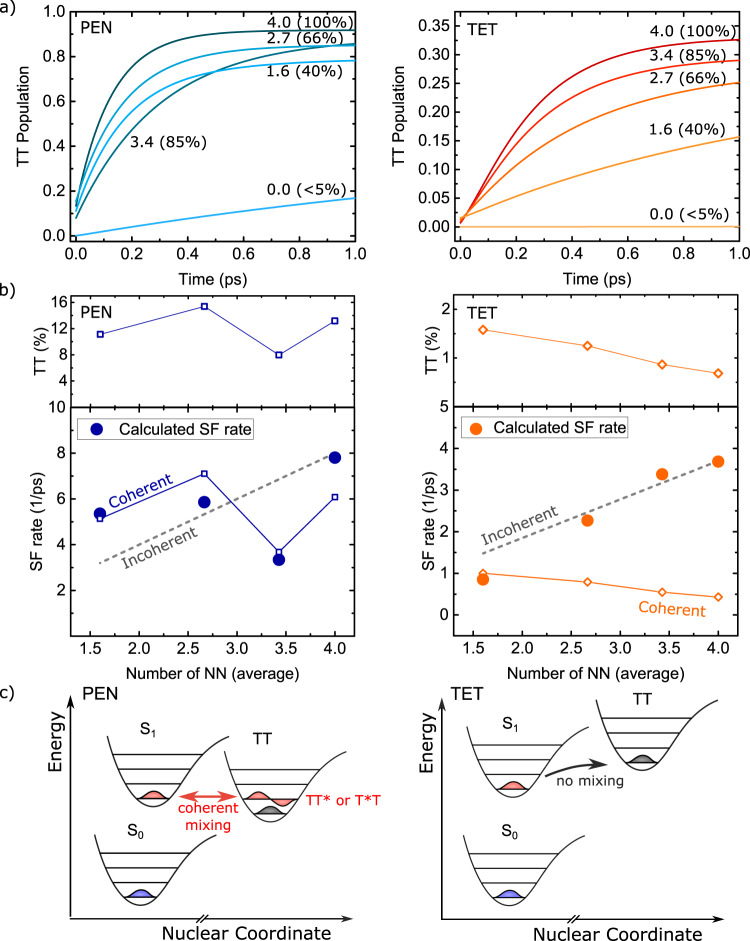
Triplet-pair-state population and SF mechanisms. **a** Calculated dynamics of the triplet-pair- (TT) state population for PEN and TET. The numbers indicate the average number of nearest neighbours (NN) and the corresponding PEN or TET fraction in the blend. **b** Top panel: TT admixture into the photoexcited singlet state. Bottom panel: Reproduction of the calculated SF rates (unscaled) from Figs. 2c, d (filled circles), shown alongside rescaled values of the TT admixture (open symbols, solid lines) and the number of nearest neighbours (dashed lines). Correlations between the SF rates and these values are indicative of the relative coherent and incoherent contributions to the dynamics, respectively. All values for zero nearest neighbours vanish for trivial reasons, see main text, and are not shown here. **c** Schematic of the two pathways of SF in PEN and TET. Due to the endothermicity of SF in TET, the triplet-pair states cannot coherently mix into the photoexcited singlet state, in contrast to PEN where such mixing is mediated by a vibrational quantum (denoted by the *).

In previous studies on neat crystals, the applied model has been extensively benchmarked against experimental data showing excellent agreement for polarisation-resolved linear absorption of TET^7^ and PEN^9^, and two-dimensional spectroscopy of PEN^52^. As shown in the Supporting Information, the model also performs well in reproducing the salient spectral changes upon the inclusion of vacancies for both PEN and TET. The calculated SF dynamics are shown in Fig. 3a. The calculations semiquantitatively reproduce the experimentally observed timescales for PEN-based blends, as well as the expected yield for neat PEN (the finite dynamics found for zero nearest neighbours is leakage caused by further-than-nearest-neighbour couplings combined with the limited crystal size). An experimental determination of the SF yields is complicated by the changes in the number of PEN molecules with changing PEN concentration, but given the marked exothermicity, experimental yields are expected to reach unity as predicted by our theory. Theoretically reaching a comparable level of agreement for TET and its derivatives has proven to be a notorious challenge with previously reported calculations yielding significant overestimates for the SF rate^17,29^. We have not addressed this challenge in the present study, reaching a similar overestimate for the SF rate; however, it is worth pointing out that a significantly lower SF rate would result from our model if the low-frequency vibrational spectrum were represented by a realistic, structured spectral density rather than a continuous Debye function. It should also be pointed out that the maximum TT population in our calculations does not exceed ~30% similarly to previous reports based on analogous modelling^17,29^, since a further depletion of S_1_ requires a triplet-separation process^39^ not included in our model, which would further skew the SF rate to longer timescales. Last, an overestimation of the electronic and vibronic coupling strengths associated with the triplet states, for which an experimental benchmark is impossible due to the triplets being optically dark, could also contribute to observed rate discrepancies. None of these factors, which are common to all theoretical treatments of TET, are expected to impact the qualitative SF trends. The favourable spectral agreement with experiment indicates that the electronic and vibronic states that dictate the energy alignments, and which underlie the coherent and incoherent couplings, are realistically reproduced, allowing us to focus on the qualitative behaviour observed for the SF rates.

Shown alongside the measured analogues in Fig. 2c,d are the calculated rates, obtained by fitting of an exponential function to the calculated evolution of the TT population (Fig. 3a) followed by a rescaling (which can be considered a correction factor accounting, e.g., for a potential mismatch in the low-energy Franck–Condon-active modes). Here it is seen that the calculations predict qualitative trends consistent with the experimental observations: a strong vacancy dependence of the SF rate for TET, and a weak dependence for PEN. As such, we have a basis to use the calculations to unravel the microscopic mechanisms underlying these trends, including those that have no direct experimental signature.

The most pronounced difference between the TET- and PEN-based blends borne out in our calculations is the degree of coherent mixing between the triplet pairs and singlet excitations. For the photoexcited singlet state S_1_, we observe an ~15% triplet-pair admixture for neat PEN, against an ~1% admixture for neat TET, as shown in Fig. 3b. Moreover, these admixtures are found to be largely independent of the vacancy concentration, being instead dependent on the energy alignment of S_1_ with the adiabatic triplet-pair state TT for which changes are only small. The decrease in admixture with increasing TET concentration is due to S_1_ and TT being held at constant energies in our calculations, for which the diabatic singlet-to-triplet-pair gap needs to be increased to offset enhanced couplings at higher concentrations, see Supporting Information for details. Interestingly, for PEN-based blends the modest fluctuations apparent in this admixture (related to finite-size effects in our calculations) are found to be faithfully followed by the calculated SF rate, which is also shown in Fig. 3b. The observed correlation between the SF rate and the degree of coherent mixing, combined with the expectation that such fluctuations will average out in extended crystals as observed in the measurements, leads us to conclude that coherence between S_1_ and TT forms the driving force for SF in PEN. This mechanism is reasonably robust against the introduction of vacancies because a single PEN neighbour can already provide a rapid SF pathway; additional PEN neighbours result in only small changes to the coherently mixed state.

In marked contrast, with coherent mixing being at least an order of magnitude weaker, SF in the TET-based blends is primarily driven by an incoherent mechanism. Consequently, the SF rate is expected to scale as the number of available neighbours for each TET molecule, which is confirmed by the strong correlation between these two quantities in Fig. 3b. Importantly, our model only describes the generation of neighbouring triplet-pair states, which explains the limited SF yield predicted by our calculations, consistent with previous findings on similar endothermic systems^30^. The subsequent triplet-separation step (and the associated gain in entropy^53^), responsible for high SF efficiencies in endothermic SF^39^, is likely to be similarly affected by the number of neighbours, since the underlying Dexter transfer mechanism is generally considered to have an incoherent nature.

Although the idea of a functional relevance of the coherence between S_1_ and TT in PEN traces back to 2011^28^, the origin of their coherent mixing has been the topic of a long-standing debate. The original hypothesis of electronic couplings between S_1_ and TT being sufficiently strong to facilitate such mixing^28^ has been contradicted by theoretical calculations^23,54–56^. Indeed, this hypothesis would be at odds with our findings showing an order-of-magnitude smaller mixing for TET, despite similar coupling strengths. More recently, various studies have suggested the presence of a vibrational resonance between S_1_ and TT to facilitate the sub-100-fs SF time constant in PEN^15,16,18^. In particular, recent theoretical work^18^ has shown an intramolecular vibrational mode with an energy of *ω*_0_ = 1150 cm^−1^ to be responsible for this vibronic enhancement. Although not emphasised at the time, from the data in that work, it can be seen that *ω*_0_ not only modulates the SF rate constant, but also the degree of admixture of triplet-pair states into S_1_.

This brings us to the important observation that the functionally important vibronic resonance generated by *ω*_0_ appears to be unique to exothermic SF, where the TT state dressed by a vibrational quantum (denoted TT*) becomes resonant with the photoexcited state S_1_, as illustrated in Fig. 3c. For endothermic SF, TT lies already above S_1_, and instead there only could be a vibronic resonance between the hot singlet state S_1_* and TT. Consistent with this idea, there is experimental evidence that SF can be accelerated by a factor of 100 from higher-lying states in TET^57^. Importantly, this principle leaves the relaxed photoexcitation S_1_ without a resonant triplet-pair state, which explains why coherent mixing is much smaller for TET than for PEN and why, consequently, SF proceeds through an incoherent mechanism instead.

We note that under idealised conditions, considering the partial transfer of excitation energy from a singlet state (donor) on one molecule to triplet-pair states (acceptor) involving a nearest neighbour, one expects the coherent SF rate to monotonically increase with the number of neighbouring PEN molecules, although far from linearly. There are several reasons why this relationship is strongly mitigated, and even destroyed, in our data. First, upon adding ever more neighbours the transfer rate will saturate. For PEN where the singlet state couples to vibrationally dressed triplet-pair states, the number of acceptor states is double the number of nearest neighbours, since the vibration can be located on either triplet-bearing molecule, which enhances the saturation effect. Second, and more importantly, the coherent transfer rate sensitively depends on the resonance between the donor singlet state and the acceptor triplet-pair states. In particular, in our calculations, where crystal sizes are limited, fluctuations of these quasi-resonant states are inevitable, giving rise to the modest rate fluctuations observed on top of what would otherwise only be a weak vacancy-dependent trend.

Last, even though structurally and energetically different from the TET blends considered here, it is interesting to note that measurements on TIPS-tetracene have indicated a partial formation of TT to occur on a sub-picosecond timescale, which has been attributed to vibrational relaxation bringing TT in full resonance with S_1_^32^. While it is conceivable that such mechanism is operative in our TET blends, it would shift the bottleneck to the subsequent triplet-separation step that remains markedly endothermic^32^, with a concomitant dependence on the number of neighbours. As such, the SF dynamics dominating the time range considered in our study will remain subject to the vacancy-dependent incoherent mechanism. To differentiate between these two endothermic mechanisms, it may be instructive to experimentally initiate SF from hot singlet states (S_1_*), which could expedite an endothermic singlet-to-triplet-conversion step but is unlikely to impact the subsequent triplet-separation event. This would, however, require spacer molecules with higher band gaps than used in the present study in order to avoid exciting the spacer molecules instead. It is also interesting to note the possibility that in the TET blends, exciton migration is convolved with the vacancy-dependent SF process, since it may allow the photoexcited singlet to find local environments with an enhanced number of neighbours. Statistically, an increase in the overall vacancy concentration is then expected to increase the necessary migration length while decreasing the migration rate. As such, we expect the behaviour discussed in the present study to be borne out in a qualitatively similar fashion.

In summary, our combined experimental and theoretical study shows that exothermic SF is susceptible to a coherent driving mechanism induced by vibrational resonances between the photoexcited singlet state and product triplet-pair states. For endothermic materials, no such vibrational resonances are possible, as a result of which SF proceeds incoherently. These mechanisms are at the core of the surprising contrast between SF in PEN-based and TET-based thin films, which are invariant to and variable against introducing spacer molecules, respectively. As such, the results obtained for these prototypical materials provide a comprehensive scenario of how SF results from the interplay of electronic energy alignment, vibronic coupling, and quantum coherence, and offer design rules for devising SF materials. For example, the desirable property of endothermicity, which enhances the energy efficiency of the process, comes at the cost of a heightened susceptibility to material defects. Exothermic SF, on the other hand, has a high degree of robustness, at the cost of excess energy deposited in vibrational modes. Hence, which scenario is more desirable depends on the energy and robustness requirements that are called for.

Progress in understanding the role of coherence in SF has been hampered by the complexity of the SF process as well as the difficulty of detecting quantum coherence. In the present study, we complement commonly used time-resolved optical measurements of SF with the controlled introduction of vacancies in both exothermic and endothermic settings, in order to fundamentally expand the number of tuneable parameters with which to interrogate the SF process. The contrasting behaviour of exothermic PEN and endothermic TET with varying vacancy concentration is a telltale of the complexity of SF, as its explanation involves the simultaneous involvement of energy alignment, vibronic coupling, and quantum coherence. The difficulty of detecting coherence has been overcome by accompanying calculations that simultaneously reproduce the key experimental observations and allow the degree of coherent mixing to be quantified. Hence, by combining comprehensive experimental control with theoretical modelling, we have followed a holistic approach that we anticipate to find repeated success in disentangling a broad range of complex photophysical processes.

## Methods

### Sample preparation

The mixed thin films of TET (Sigma Aldrich, 99.99%) and 6PH (Lambson Japan Co. Ltd. 99%) or PIC (Tokyo Chemical Industry Co. Ltd. 99.9%) were grown by organic molecular beam deposition on silicon with a native oxide layer and on borofloat glass substrates at a base pressure of 1 × 10^−9^ mbar. The total growth rate was 0.6 nm/min, with the rates of the two materials monitored separately by two quartz crystal microbalances, calibrated using X-ray reflectivity. Unless noted otherwise, the final film thickness was 80 nm.

### Structural and optical characterisation

X-ray reflectivity was measured on a diffractometer (3303TT, GE) using Cu Kα radiation (λ = 1.5406 Å) and a 1D detector (Meteor 1D, XRD Eigenmann). Grazing-incidence X-ray diffraction and the reciprocal space maps were measured at beamline SixS at Soleil, Gif-sur-Yvette, France, using a wavelength of λ = 0.9538 Å and on a Xeuss 2.0 SAXS/WAXS system (Xenocs) with a Dectris Pilatus3R 300-K detector (λ = 1.5406 Å). The sample was kept in vacuum during the measurements to avoid beam damage.

UV–vis transmission spectra were measured using a Perkin Elmer Lambda 950 spectrophotometer. Photoluminescence-excitation spectroscopy was performed on a Horiba Fluorolog-3 DF spectrofluorimeter using a 450-W xenon lamp for excitation and a Hamamatsu R2658P PMT to monitor the emission.

### Time-resolved photoluminescence

Time-resolved photoluminescence measurements were taken with a Hamamatsu C4334 Streakscope having a time resolution of 20 ps and a spectral resolution of 2.5 nm. The 800-nm output of an 80-MHz Coherent Vitesse Ti:Sapphire oscillator was frequency doubled to generate the 400-nm excitation pulse. A Pockels cell controlled by a ConOptics pulse-picking system was used to adjust the repetition rate of the oscillator to 100 kHz. A 450-nm-long wave-pass filter was placed before the streak camera to minimise the contribution of laser scatter to the signal. All measurements were performed in a vacuum cryostat (10^−5^ torr) fitted with optical windows, and pulse fluences remained below 1.2 μJ/cm^2^.

### Modelling

Crystalline tetracene and pentacene were parameterised based on previous theoretical studies^7,9,18^. A full microscopic basis was used including for each acene molecule, a (diabatic) singlet ground, and a singlet excited state, as well as a cationic, an anionic, and a first triplet state. Moreover, a single quantum harmonic oscillator was included for each molecule to account for linear coupling of the electronic transitions to a single intramolecular vibration by means of the Holstein Hamiltonian. The quantum dynamics was obtained by applying non-Markovian Redfield theory in the secular approximation using a Debye spectral density to account for the Holstein-coupled vibrational modes other than the quantum harmonic oscillator. The noninteracting spacer molecules were accounted for by taking out acene molecules and their associated electronic states from the microscopic basis set.

## Supplementary information

Supplementary Information

## Data Availability

The experimental data generated in this study have been deposited in the figshare database (10.6084/m9.figshare.14916000) and are also available on request from K.B. and R.T.

## Code availability

The code used for the calculations presented in this work is available at https://github.com/tempelaar-team/VibroNISE4.
