# Supplementary material for: Vacancy control in acene blends links exothermic singlet fission to coherence
Source: Nat Commun. 2021 Aug 26;12:5149. doi: 10.1038/s41467-021-25395-9 (PMC8390483; doi:10.1038/s41467-021-25395-9)
Supplement: Supplementary file 1 — Supplementary Information [file 41467_2021_25395_MOESM1_ESM.pdf]

Supporting Information for "Vacancy control in acene blends links exothermic singlet fission to coherence"

Clemens Zeiser[1], Chad Cruz[2], David R. Reichman[3], Michael Seitz[4], Jan Hagenlocher[1], Eric L. Chronister[5], Christopher J. Bardeen[2], Roel Tempelaar[3,6], and Katharina Broch[1]

[1]Institute of Applied Physics, University of Tübingen, Auf der Morgenstelle 10, 72076 Tübingen, Germany
[2]Department of Chemistry, University of California at Riverside, 501 Big Springs Road, Riverside, CA 92521, USA
[3]Department of Chemistry, Columbia University, 3000 Broadway, New York, NY 10027, USA
[4]Institute of Inorganic Chemistry, University of Tübingen, Auf der Morgenstelle 18, 72076 Tübingen, Germany
[5]University of Nevada, Las Vegas, 4505 S. Maryland Pkwy., Las Vegas, NV 89154, USA
[6]Department of Chemistry, Northwestern University, 2145 Sheridan Rd, Evanston, IL 60208, USA

Contents

1. Structural properties 2
2. Optical characterization of TET:6PH and TET:PIC blends 4
    2.1 Gaussian fit model of the absorption spectra 5
    2.2 Decomposition of the PL spectra 6
    2.3 TRPL traces of TET:6PH and TET:PIC blends 8
    2.4 Monoexponential fits of the TRPL traces 9
    2.5 Magnetic field dependence of the TRPL traces 10
    2.6 Photoluminescence excitation spectroscopy 10
3. Theory 11
    3.1. Theoretical model 11
    3.2. Parametrization of pentacene 12
    3.3. Parametrization of tetracene 12
    3.4 Comparison of calculated absorption spectra with the experimental data 13
References 13

1. Structural properties

Figures S1a and S1b show the X-ray reflectivity (XRR) scans, from which the mixing ratio-dependent out-of-plane lattice spacings of the TET blends shown in the main text have been extracted by analysing the Bragg preak positions. Since TET and PIC molecules have approximately the same length, no significant shift in the Bragg peak position is observed between 50% TET and neat PIC in contrast to the TET:6PH blends. From the absence of Bragg peaks of neat TET phases in blends with 50% TET concentration or less in TET:PIC blends and 45% TET or less in TET:6PH blends we can exclude a phase separation of TET for these TET concentrations. For blends of 50% TET or higher, Bragg peaks corresponding to the bulk phase of TET[1] can be observed in TET:6PH and TET:PIC blends, indicating limited intermixing and a phase separation of neat TET and a mixed phase.

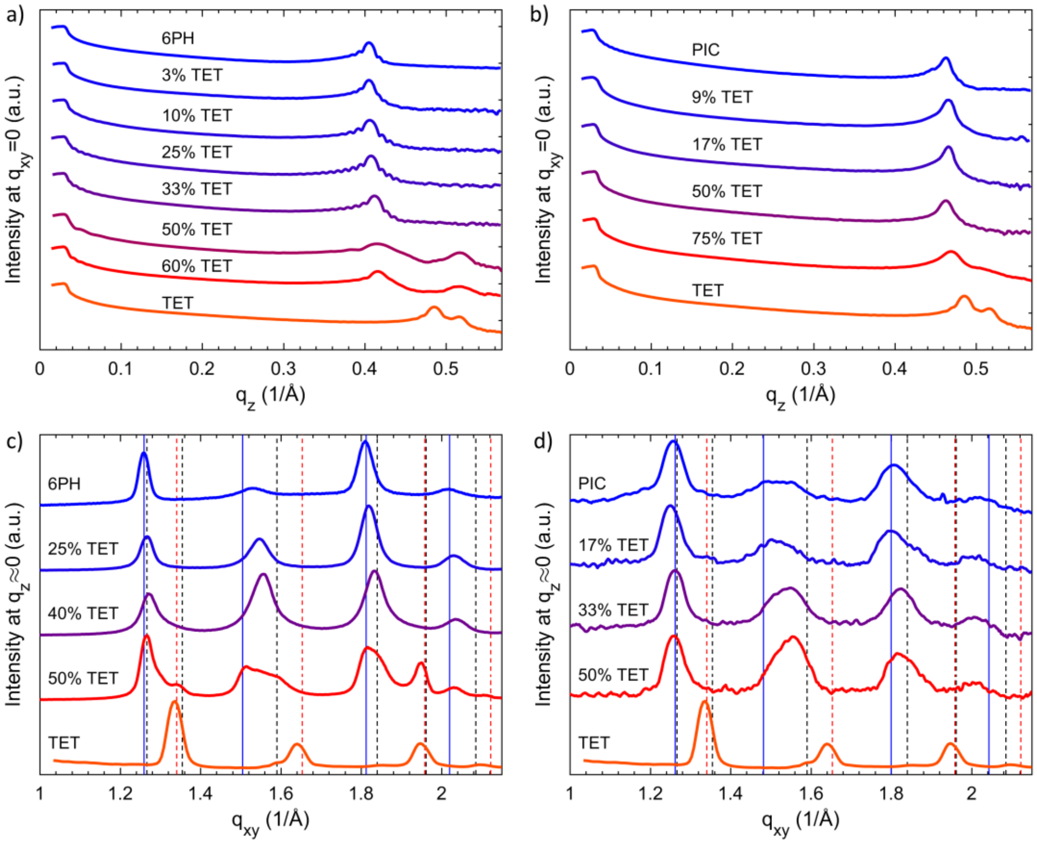

Figure S1: (a,b) XRR scans on 80 nm TET:6PH (a) and TET:PIC blends (b). (c,d) Grazing incidence X-ray diffraction scans of 40 nm TET:6PH and 80 nm TET:PIC blends. Blue vertical lines indicate the position of in-plane Bragg peaks corresponding to neat 6PH (c) or neat PIC (d) films.[2,3] The Bragg peak positions shown for 6PH are based on the chrysene unit cell as discussed in the text. Dotted vertical lines correspond to Bragg peaks of the bulk (black) and the thin film (red) phase of TET.[1]

Grazing incidence X-ray diffraction data are shown in Figures S1c and S1d. For TET concentrations below the critical concentration for limited intermixing, the positions of the in-plane Bragg peaks in both mixed systems are close to the corresponding reflexes of the respective spacer compound with only slight shifts in position. Furthermore, no TET Bragg peaks are visible, supporting the conclusion of complete intermixing for concentrations below 45% (TET:6PH) and 50% (TET:PIC), respectively. In contrast, for the TET:6PH blend with 50% TET concentration, we observe the appearance of TET Bragg peaks in complete agreement with the scenario of limited intermixing.

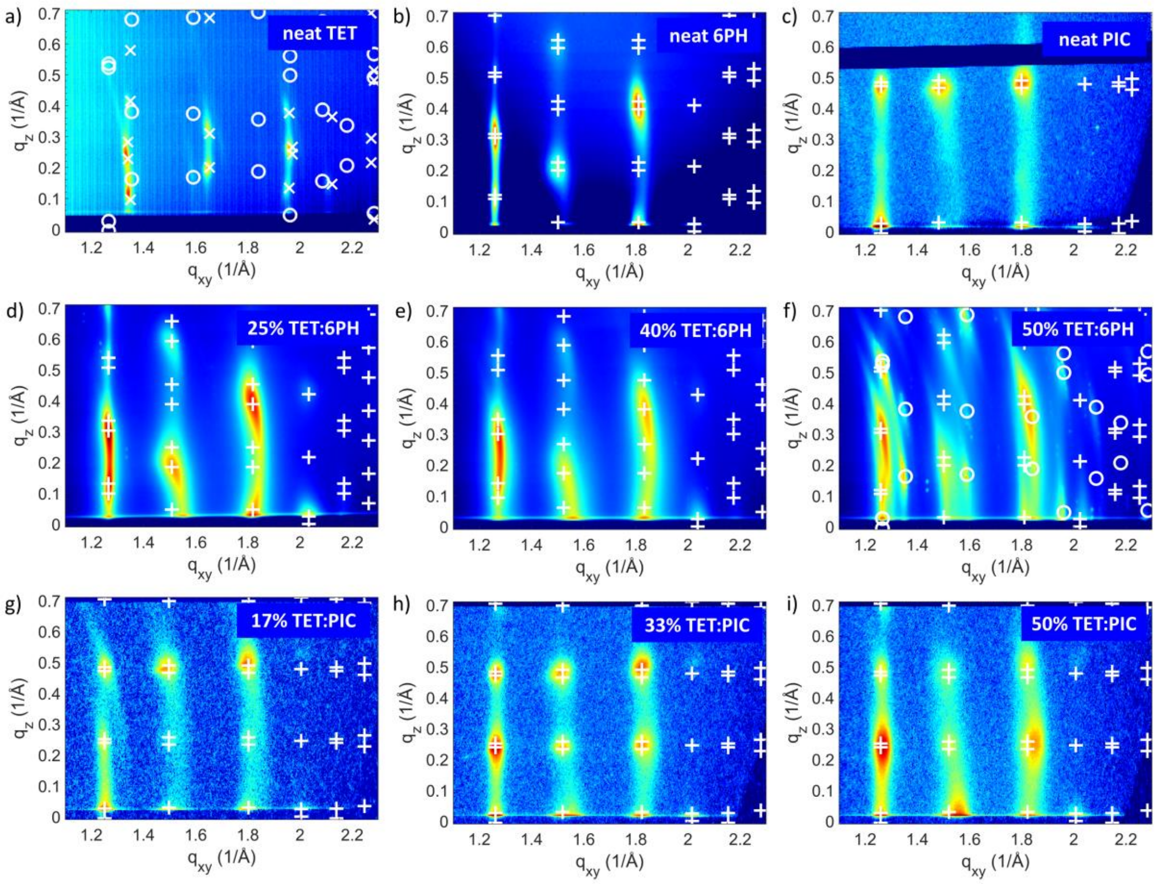

Figure S2: Reciprocal space maps of thin films measured in grazing incidence geometry. (a-c) 20 nm TET, 20 nm 6PH, and 80 nm PIC thin films. (d-f) 40 nm TET:6PH blends. (g-i) 80 nm TET:PIC blends. The white markers show calculated Bragg-peak positions. Circles correspond to Bragg-peaks of the bulk-phase, and crosses to the thin film-phase of TET[1]. Plus symbols are calculated based on unit cells according to Table S1.

Figure S2 shows reciprocal space maps of neat films and the blends. Two polymorphs are known for TET[1] and at a substrate temperature of 240 K we find neat TET to grow well-ordered, standing upright on native silicon with its unit cell corresponding to the polymorph with larger lattice spacing, see Figure S2a. Note that all other films have been grown at a substrate temperature of 300 K. The calculated reflections shown in Fig. S2b and S2c for 6PH and PIC have been optimized based on reported unit cell parameters[2,3], see Table S1 and are consistent with the thin film structures[2,4]. In the reciprocal space maps of the TET:PIC blends, strong reflections occur at 0.25 $Å^{-1}$ (Figure S2g-i), which are weak or absent for neat PIC[3], but can be explained by a PIC unit cell with a c-axis that is twice as long as that of the unit cell in neat PIC films and has 4 instead of 2 molecules but has the same symmetry and in-plane parameters. This should not affect the photophysics, which are dominated by intermolecular interactions within the a-b plane.

Comparing the unit cell parameters of the TET:spacer blends with that of the respective spacer compound, we observe a slight, continuous increase of approximately 0.15 Å of the longer in-plane axis. The change in the shorter in-plane axis is within the errorbars in both cases. The shift is indicative of the formation of a solid solution, in which lattice sites are occupied statistically by either TET or spacer molecules. At a TET concentration of 50%, reflections from both a mixed phase and a neat TET phase (see plus symbols and circles in Figure S2f) can be observed, indicating limited intermixing as discussed for Figure S1 and in the main text. Since the investigation of the optical properties is

complicated by this phase coexistence, we focused our experimental analysis on the samples with TET concentrations below 50%.

Table S1: Unit cell parameters of neat films and blends determined based on previous reports[2-4]. The S2 labelled column indicates in which panel of Figure S2 calculated reflections of the respective unit cell are shown as plus symbols. Note that the a and b parameters (and accordingly the angles α and β) of 6PH and PIC have been switched compared to the original publications[2-4] for comparability.

**Unit cell**	**Fig. S2**	**a (Å)**	**b (Å)**	**c (Å)**	**α (°)**	**β (°)**	**γ (°)**
neat 6PH	b,f	6.22 ± 0.1	8.37 ± 0.1	32.0 ± 0.1	98.0 ± 2	90.0 ± 2	90.0 ± 0.5
25% TET:6PH	d	6.18 ± 0.1	8.33 ± 0.1	31.0 ± 0.1	96.5 ± 2	90.0 ± 2	90.0 ± 0.5
40% TET:6PH	e	6.17 ± 0.1	8.25 ± 0.1	30.5 ± 0.1	96.0 ± 2	90.0 ± 2	90.0 ± 0.5
neat PIC	c	6.23 ± 0.1	8.37 ± 0.1	13.5 ± 0.1	90.5 ± 2	90.0 ± 2	90.0 ± 0.5
17% TET:PIC	g	6.27 ± 0.1	8.39 ± 0.1	27.0 ± 0.1	90.5 ± 2	90.0 ± 2	90.0 ± 0.5
33% TET:PIC	h	6.24 ± 0.1	8.27 ± 0.1	27.0 ± 0.1	90.5 ± 2	90.0 ± 2	90.0 ± 0.5
50% TET:PIC	i	6.27 ± 0.1	8.22 ± 0.1	27.0 ± 0.1	90.5 ± 2	90.0 ± 2	90.0 ± 0.5

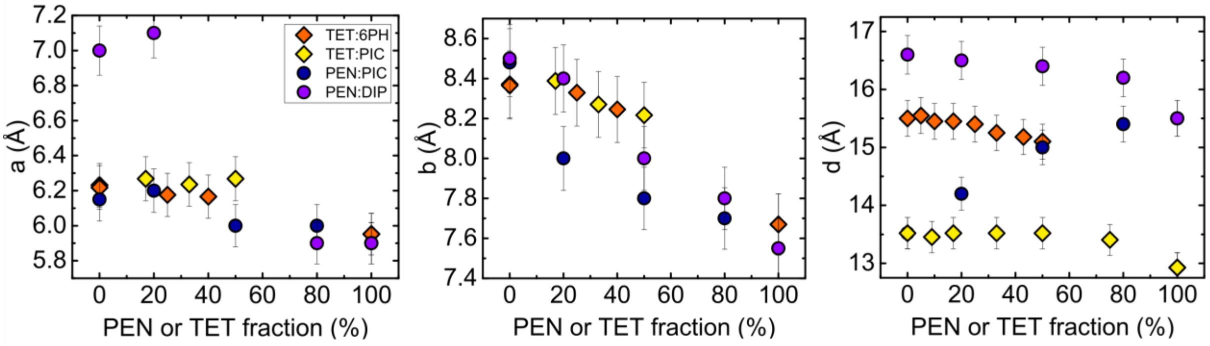

Figure S3: Summary of unit cell parameters for the two TET blends studied in this work and the PEN blends (Ref. 5) used for comparison.

2. Optical characterization of TET:6PH and TET:PIC blends

2.1 Gaussian fit model of the absorption spectra

For a quantitative analysis of the absorption spectra, they were fitted with a gaussian model, see Figure S4. For blends with TET concentrations > 17% the Davydov splitting can be clearly seen in both systems. The analysis also reveals the transition to a spectrum dominated by monomeric TET for blends with TET concentrations < 17%.

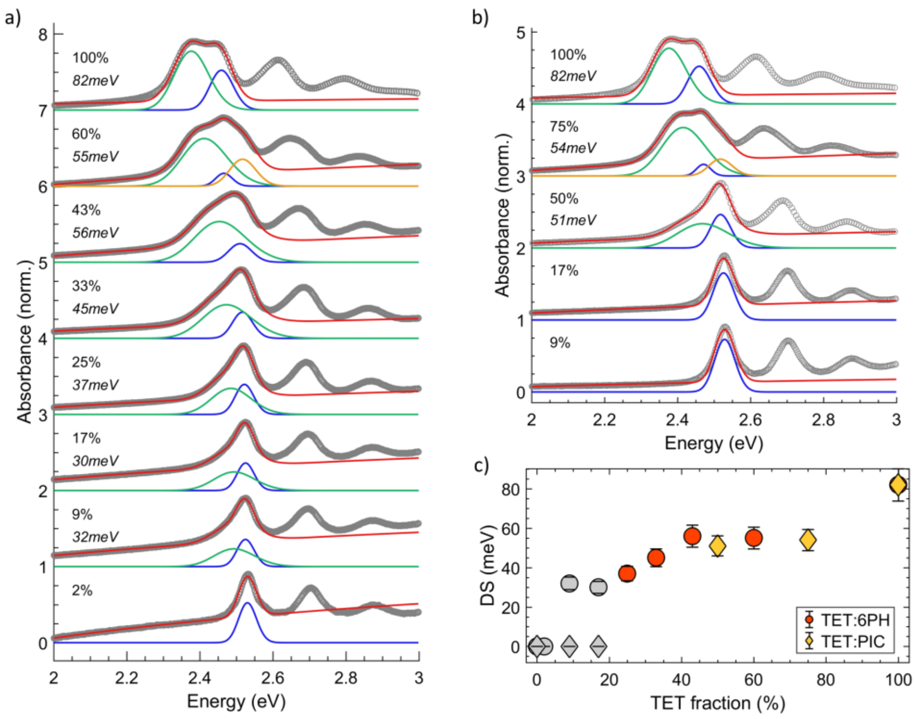

Figure S4: Fits of the absorption spectra of (a) TET:6PH and (b) TET:PIC blends with a model of three gaussians and a first-order polynomial. For each spectrum, the TET concentration is given in % and the difference in position of the blue and the green gaussian, corresponding to the Davydov splitting (DS), is given in meV. (c) DS extracted from the fit shown in panels (a) and (b). Grey symbols indicate values extracted from absorption spectra where the assignment of the energetically lowest transitions to a DS is difficult or where the DS can no longer be observed.

2.2 Decomposition of the PL spectra

In a herringbone structure, as formed in neat TET films and in the blends, a given TET molecule has four nearest neighbours. Due to the statistical occupation of lattice sites, these nearest neighbour sites can be occupied by 0 to 4 TET molecules with a probability given by a binomial distribution and depending on the TET concentration. The five different configurations, referred to C0 - C4 in the following, are shown in the inset of Figure S5.

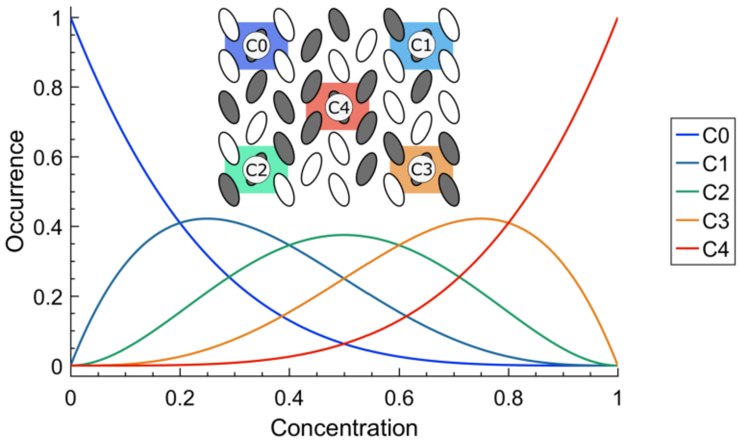

Figure S5: Probability of configurations C0 to C4 in solid solutions following a binomial distribution. The five configurations are shown in the inset.

For the decomposition of the PL spectra we fitted the data to a superposition of five contributions, see Figure S6 and S7, with a relative weight C, which depends on the TET fraction *f*,

$$I_{\mathrm{PL}}(f,\lambda) = C_0(f)\cdot F_0(\lambda) + C_4(f)\cdot F_4(\lambda) + C_1(f)\cdot F_{\mathrm{b}}(\lambda - \Delta\lambda_1) + C_2(f)\cdot F_{\mathrm{b}}(\lambda - \Delta\lambda_2) + C_3(f)\cdot F_{\mathrm{b}}(\lambda - \Delta\lambda_3)$$

The first contribution is the monomer spectrum $F_0(\lambda)$, taken from the blend with 2% TET (0 TET neighbours), and the second contribution is the bulk spectrum $F_4(\lambda)$ of a neat TET film (4 TET neighbours). Motivated by the modest changes of the spectral shape between different blends other than those with very small TET concentration, we assumed the PL spectra of the other three possible contributions (1–3 TET neighbours) to have the shape of the bulk spectrum, but displaced in wavelength by an amount $\Delta\lambda_i$ (i = 1,2,3) which was taken as a global fit parameter. Fitting the concentration-dependent PL spectra to the superposition of these five contributions allowed us to determine their relative weights C shown in Figures S6 and S7 and Figure 1 of the main text.

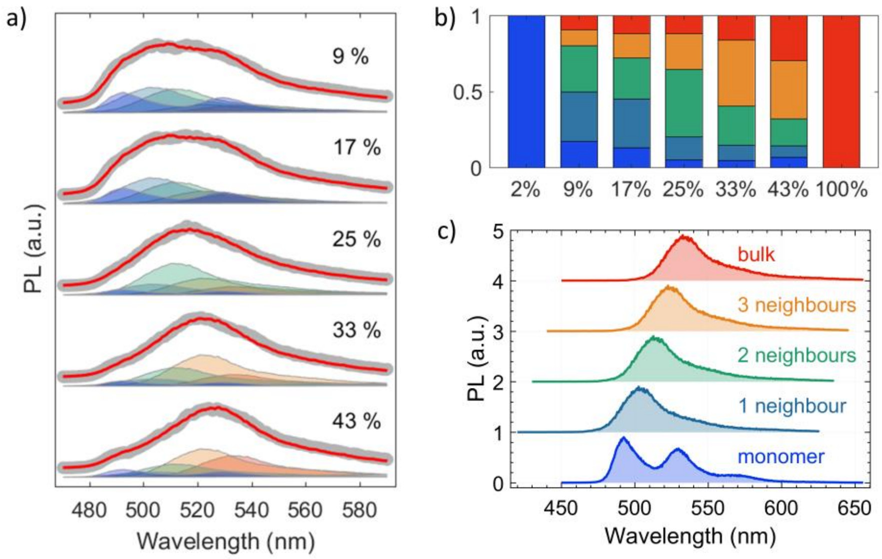

Figure S6: Decomposition of the PL spectra of TET:6PH blends into PL spectra of five configurations (0 to 4 TET neighbours of a TET molecule). a) Fits (red) of the experimental PL spectra (grey) by a superposition of the PL spectra of the five contributions as described in this section. b) Relative contributions of the individual PL spectra to the fitted PL spectrum. For a reasonable relative contribution relating the monomer to the bulk spectrum, the spectra have been normalised accounting for areas of gaussians fitted to the main peak of the respective spectrum. c) PL spectra of the different configurations. The monomer and bulk spectrum are those of the 2% TET blend and the neat TET film, respectively. The PL spectra of the other three configurations correspond to the bulk spectrum shifted in wavelength position.

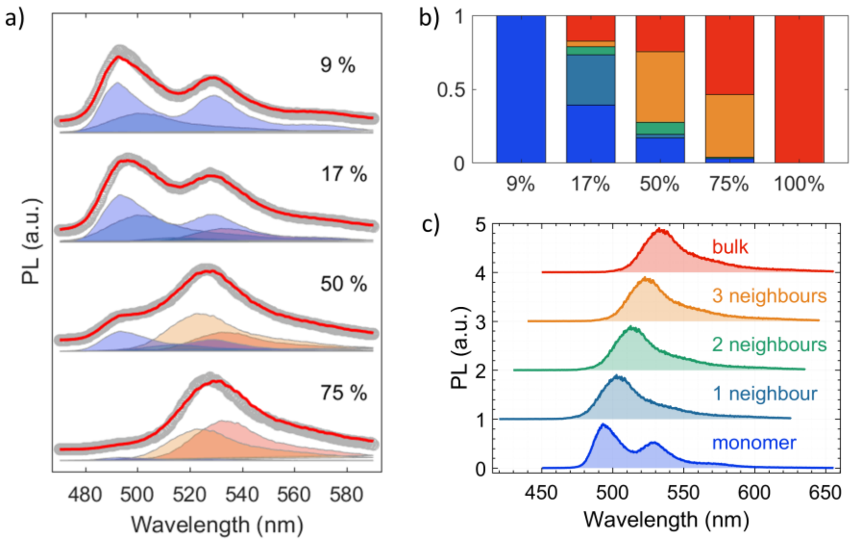

Figure S7: Decomposition for TET:PIC blends as described in the figure caption of Figure S6 for TET:6PH. Contributions from configurations of 2 or 3 TET neighbours cannot be easily distinguished, which might explain the weak contribution of the configuration of 2 TET neighbours to the spectral decomposition.

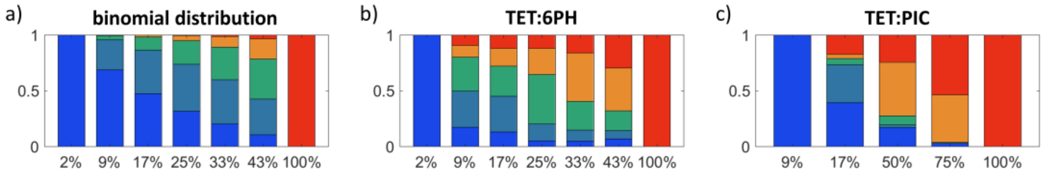

Figure S8: (a) Contributions according to a binomial distribution as shown in Figure S5. (b) Decomposition of the PL spectra of TET:6PH blends as shown in Figure S6. (c) Decomposition of the PL spectra of TET:PIC blends as shown in Figure S7.

The results of the decomposition of the PL spectra can be compared to a binomial distribution, see Figure S8. Since the relative weight of a contribution in the PL spectrum depends also on the fluorescence lifetime, which varies with the number of TET neighbours, one would first expect a deviation of the PL decompositions from a binomial distribution and a larger contribution of those configurations with long fluorescence lifetimes, i.e. few TET neighbours. Interestingly, this effect does not occur when looking at the experimental decompositions (Figure S8 b,c), but rather the opposite, as we observe in both systems a larger contribution of sites with 2−4 TET neighbours than statistically expected. Since we can exclude phase-separated TET crystallites based on X-ray diffraction, this deviation points towards migration of excitons towards locations where an increased number of nearest neighbors for a given TET molecule lowers the local energy by intermolecular interactions.

2.3 TRPL traces of TET:6PH and TET:PIC blends

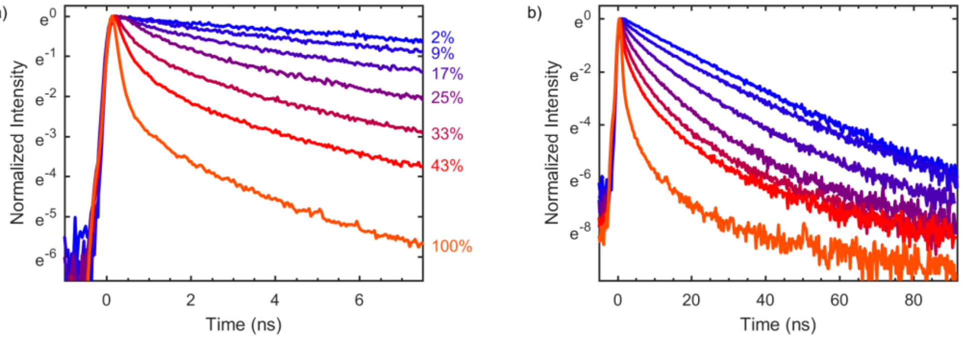

Figure S9: TRPL traces of TET:6PH blends in two different time windows.

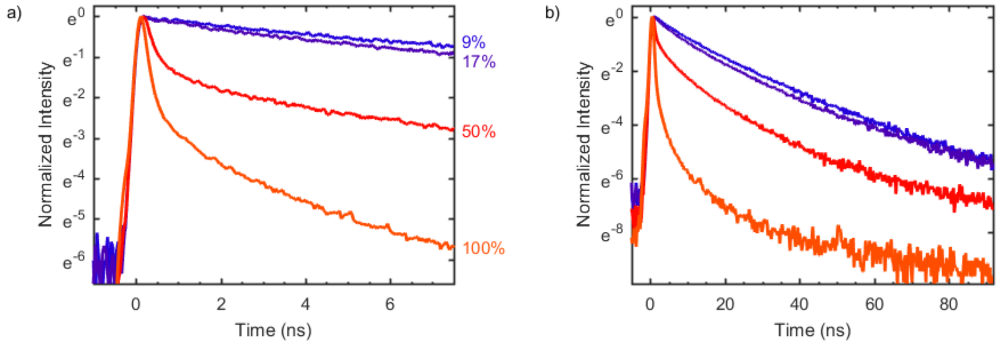

Figure S10: TRPL traces of TET:PIC blends in two different time windows.

2.4 Monoexponential fits of the TRPL traces

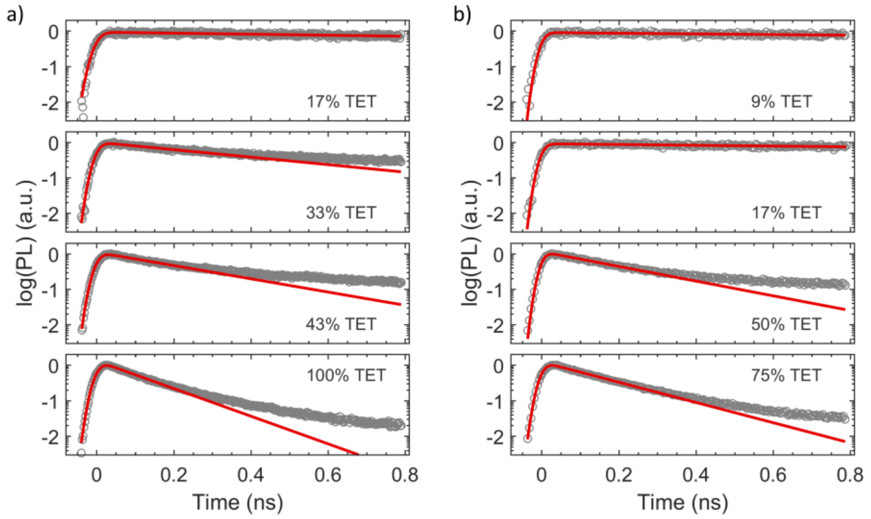

Figure S11: Comparison of fits and experimental data for representative TRPL traces of TET blends ((a) TET:6PH, (b) TET:PIC) optimized for short times, i.e. the fastest SF rates in the respective blend. The TRPL traces show multiexponential dynamics due to different configurations in each blend as shown in the decomposition of the PL spectra.

The normalized TRPL traces were fitted using a convolution *F(t)* of a Gaussian instrument response function *g (t)* and a monoexponential decay *f(k,t)* with amplitude *A* and the rate *k* as fitting parameters:

$$F(t) \; = \; A \cdot \int f(k,t') \; \cdot g(t-t') \, dt'$$

$$f(k,t) = \begin{cases} \exp(-k \cdot t) & \text{for } t \geq 0 \\ 0 & \text{for } t < 0 \end{cases}$$

$$g(t) \; = \; \exp\left(-\left(\frac{t}{\tau}\right)^2\right), \;\; \tau = 20 \text{ ps}$$

The rates as shown in Figure 2c in the main text are summarized in Tab. S2.

Table S2: Fit results.

**TET:6PH**												
**% TET**	2	5	9	10	17	25	31	33	40	43	50	100
**k (ps^{-1})**	0.023	0.129	0.276	0.171	0.331	1.291	2.177	2.434	3.712	4.302	5.017	8.948
**TET:PIC**												
**% TET**				10	16						49	100
**k (ps^{-1})**				0.219	0.252						4.777	8.948

2.5 Magnetic field dependence of the TRPL traces

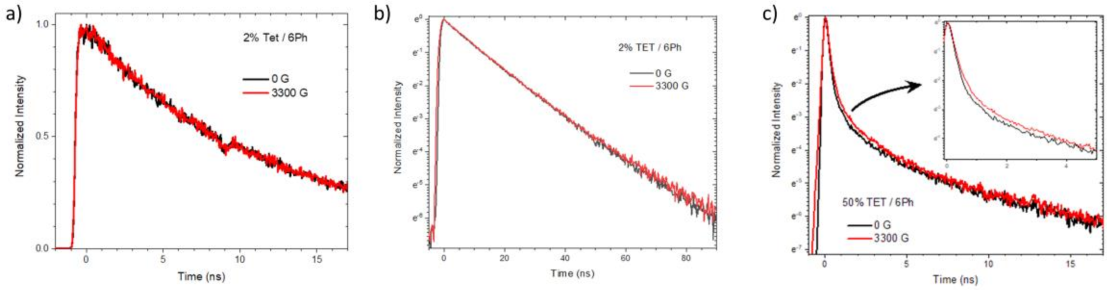

Figure S12: Influence of an external magnetic field on the TRPL signal of a TET:6PH (2% TET) blend in two different time windows (a,b) and a 50% TET blend (c). Shown are signals at a magnetic field of 0 G (black line) and at 3300 G (red line).

2.6 Photoluminescence excitation spectroscopy

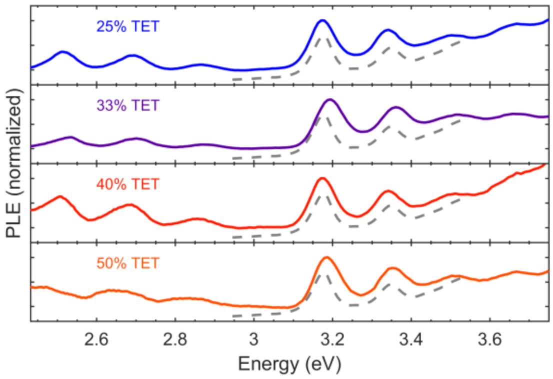

Figure S13: Photoluminescence excitation spectroscopy measurements of TET:6PH blends, demonstrating a lack of energy transfer from 6PH to TET for excitation energies below 3.1 eV (λ = 400 nm). This justifies excluding this energy transfer pathway in our analysis of the SF rates of the blends. The absorption spectrum of 6PH is shown as a dashed grey line for comparison.

3. Theory

3.1. Theoretical model

The theoretical model applied in this study has for the most part been explained in detail in Refs. 6 and 7. In short, the model describes molecular crystals using a diabatic electronic basis involving for each molecule a singlet ground state (s_0), singlet excited state (s_1), cationic state (c), anionic state (a), and a lowest-lying triplet state (t). The calculations of the singlet fission dynamics and excited state properties of the crystal were performed in the electronic manifold consisting of single singlet excitations, pairs of charged excitations, and pairs of triplet excitations, such that all states have a total spin of zero, and are charge neutral. Both pentacene and tetracene crystals were considered as two-dimensional sheets of herringbone-stacked molecules in the crystallographic ab plane, neglecting the weak electronic couplings along the c direction.

The electronic basis was complemented by a series of quantum harmonic oscillator eigenstates for each molecule, allowing to quantum-mechanically describe a single, intramolecular vibrational mode, denoted ω_0. In doing so, we restricted ourselves to the two-particle approximation[8,9], limiting the total number of vibrationally and/or electronically excited molecules to two. The basis was further truncated by imposing a total number of vibrational quanta, as well as truncation radii for the distance between charge pairs, triplet pairs, and between ground state vibrations and singlet excitations. Using this vibronic basis, we evaluated the Holstein Hamiltonian, given by[6]

$$\hat{H} = \hat{H}_{\mathrm{el}} + \omega_0 \sum_n \hat{b}_n^\dagger \hat{b}_n + \omega_0 \sum_{x=\mathrm{s}_1,\mathrm{c},\mathrm{a},\mathrm{t}_1} \sum_n \lambda_x \left(\hat{b}_n^\dagger + \hat{b}_n + \lambda_x\right) |(x)_n\rangle\langle(x)_n|.$$

Here, $\hat{H}_{\mathrm{el}}$ denotes the electronic Hamiltonian including the electronic transition energies, dipole-dipole couplings, and electron- and hole-transfer couplings, and is explicitly given by Eqs. 3-7 of Ref. 6. The second term of $\hat{H}$ accounts for the vibrational energy associated with the intramolecular mode ω_0, where $\hat{b}_n^{(\dagger)}$ represents the vibrational annihilation (creation) operator acting on molecule n. The last term of $\hat{H}$ describes bilinear (Holstein) coupling of this mode to each of the local electronic states at molecule n, denoted $(x)_n$ (with $x = \mathrm{s}_1$,c,a,t_1), where the coupling strength is quantified by λ_x. The Holstein Hamiltonian was numerically diagonalized, yielding (adiabatic) eigenvectors and eigenenergies. The photoexcited singlet state, S_1, was identified as the lowest-energy eigenstate with a dominant admixture of diabatic singlet excitations. The triplet pair admixture into this state was calculated as the expectation value $\sum_i |\langle S_1 | (\mathrm{tt})_i \rangle|^2$, where i labels the diabatic triplet pairs, $(\mathrm{tt})_i$.

The non-interacting spacer molecules were accounted for by taking molecules (and their associated basis states) out of the crystal. This was done such that the average distance between vacancies is maximized. The average number of neighbouring molecules was calculated as $1/(N) \sum_n \eta_n$ where n runs over the total of N remaining molecules, and η_n counts the number of neighbours of molecule n. The linear absorption spectra shown in Fig. S15 were calculated using Fermi's golden rule based on 4×4 unit cells (up to 32 molecules) for PEN and 2×2 unit cells (up to 8 molecules) for TET. Consistent with previous studies[6,10-12] we did not include energetic disorder in the Hamiltonian, although noting that this factor is worthy of consideration in follow-up studies, and the absorption line broadening is treated entirely phenomenologically.

The dynamics were calculated using Markovian Redfield theory in the secular approximation, representing vibrational modes other than ω_0 by a Debye spectral density, while truncating the Redfield tensor to the eigenstates with energies lower than a threshold value which was increased until

convergence was reached. For both PEN and TET crystals, dynamical calculations were limited to 2×2 unit cells (up to 8 molecules) to keep computations manageable.

3.2. Parametrization of pentacene

For the parametrization of crystalline pentacene applied in this study we refer to Refs. 6 and 7, while noting that we have specifically applied the parameters associated with the mode frequency $\omega_0 = 1150\ \mathrm{cm}^{-1}$. Reproduced in Tab. S3 are the nearest-neighbour HOMO−HOMO, LUMO−LUMO, and HOMO−LUMO electron transfer integrals, adapted from Refs. 6 and 14-16, which were obtained by means of density functional theory using the B3LYP functional and a double zeta basis set.

3.3. Parametrization of tetracene

The parametrization applied for crystalline tetracene is largely identical to those reported by Yamagata *et al.* for the same system[10]. In reporting these parameters, we follow the same conventions and definitions as used for crystalline pentacene in Refs. 6 and 7. Moreover, all parameters not specified here were taken to be the same as those for crystalline pentacene in Refs. 6 and 7. The lattice constants were taken to be 7.84 Å and 6.06 Å along the crystallographic a and b directions, respectively, while an angle of 85.8° was used between the a and b lattice vectors. The molecular (diabatic) $\mathrm{s}_0 - \mathrm{s}_1$ and $\mathrm{s}_0 - \mathrm{tt}$ transition energies were adjusted for each vacancy configuration so that the adiabatic S_1 and TT energies coincide with 19460 cm^{-1} and 19945 cm^{-1}, respectively[11]. The quantum vibrational mode was parametrized as $\omega_0 = 1430\ \mathrm{cm}^{-1}$, with $\lambda_{\mathrm{s}_1}^2 = 1.00$, $\lambda_{\mathrm{a}}^2 = 0.45$, $\lambda_{\mathrm{c}}^2 = 0.32$, and $\lambda_{\mathrm{t}}^2 = 1.00$[6,12,13]. As in previous work[10], couplings between local charge transfer configurations were neglected. Dipole-dipole couplings between local $\mathrm{s}_0 - \mathrm{s}_1$ transitions were included using the values reported in Fig. 2 of Ref. 10 and screened by a dielectric constant $\varepsilon = 3$. The local charge transfer state energies (corresponding to $\varepsilon = 3$) as well as the HOMO−HOMO, LUMO−LUMO, and HOMO−LUMO electron transfer integrals were taken from the same reference, of which nearest-neighbour values are reproduced in Tab. S3. These couplings were obtained through density functional theory at the B3LYP/TZVP level.

Table S3: Calculated nearest-neighbour electronic couplings of PEN and TET crystals adapted from Refs. 6, 10, 14-16. Values shown are given in units of cm^{-1}.

	$(\boldsymbol{\Delta a}, \boldsymbol{\Delta b})$	HOMO−HOMO	LUMO−LUMO	HOMO−LUMO
**PEN**	$(1/2, 1/2)$	-835	-791	-737
	$(-1/2, -1/2)$	-835	-791	594
	$(1/2, -1/2)$	507	808	-682
	$(-1/2, 1/2)$	507	808	600
**TET**	$(1/2, 1/2)$	203	583	-355
	$(-1/2, -1/2)$	203	583	479
	$(1/2, -1/2)$	-610	-564	-480
	$(-1/2, 1/2)$	-610	-564	367

3.4 Comparison of calculated absorption spectra with the experimental data

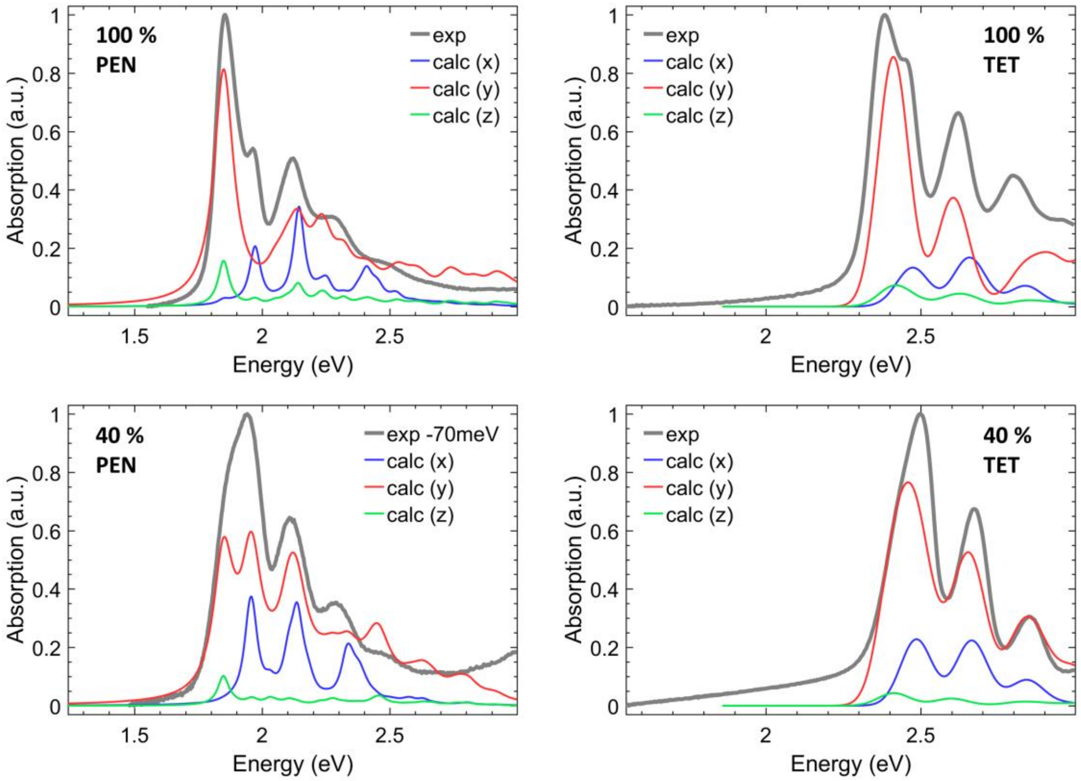

Figure S14: Comparison of polarization-resolved calculated absorption spectra (blue, red and green lines) with the experimental data (dark grey).

In order to confirm the accuracy of our model, we calculated the absorption spectra for light polarized linearly along the three spatial coordinates. Figure S14 shows the excellent agreement of the calculated and experimentally determined spectra.

References

1. Nahm, R. K. & Engstrom, J. R. Who's on first? Tracking in real time the growth of multiple crystalline phases of an organic semiconductor: Tetracene on SiO_2. *J. Chem. Phys.* **146**, 52815 (2017).
2. Krygowski, T. M., Ciesielski, A., Swirska, B. & Leszczynski, P. Variation of molecular-geometry and aromatic character of chrysene and perylene in their eda complexes-refinement of x-ray crystal and molecular-structure of chrysene and perylene. *Pol. J. Chem.* **68**, 2097–2107 (1994).
3. De, A., Ghosh, R., Roychowdhury, S. & Roychowdhury, P. Structural analysis of picene, $C_{22}H_{14}$. *Acta Crystallogr. C* **41**, 907–909 (1985).
4. Zwadlo, M. *et al.* Structure of Thin Films of [6] and [7]Phenacene and Impact of Potassium Deposition. *Adv. Opt. Mater.* 2002193 (2021).
5. Broch, K. *et al.* Robust singlet fission in pentacene thin films with tuned charge transfer interactions. *Nat. Commun.* **9**, 954 (2018).
6. Tempelaar, R. & Reichman, D. R. Vibronic exciton theory of singlet fission. I. Linear absorption and the anatomy of the correlated triplet pair state. *J. Chem. Phys.* **146**, 174703 (2017).
7. Tempelaar, R. & Reichman, D. R. Vibronic exciton theory of singlet fission. III. how vibronic

coupling and thermodynamics promote rapid triplet generation in pentacene crystals. *J. Chem. Phys.* **148**, 244701 (2018).
8. Philpott, M. R. Theory of the coupling of electronic and vibrational excitations in molecular crystals and helical polymers. *J. Chem. Phys.* **55**, 2039–2054 (1971).
9. Spano, F. C. Absorption and emission in oligo-phenylene vinylene nanoaggregates: The role of disorder and structural defects. *J. Chem. Phys.* **116**, 5877–5891 (2002).
10. Yamagata, H. *et al.* The nature of singlet excitons in oligoacene molecular crystals. *J. Chem. Phys.* **134**, 204703 (2011).
11. Morrison, A. F. & Herbert, J. M. Evidence for singlet fission driven by vibronic coherence in crystalline tetracene. *J. Phys. Chem. Lett.* **8**, 1442–1448 (2017).
12. Ito, S., Nagami, T. & Nakano, M. Density analysis of intra-and intermolecular vibronic couplings toward bath engineering for singlet fission. *J. Phys. Chem. Lett.* **6**, 4972–4977 (2015).
13. Tempelaar, R. & Reichman, D. R. Vibronic exciton theory of singlet fission. II. two-dimensional spectroscopic detection of the correlated triplet pair state. *J. Chem. Phys.* **146**, 174704 (2017).
14. Beljonne, D., Yamagata, H., Brédas, J. L., Spano, F. C. & Olivier, Y. Charge-transfer excitations steer the davydov splitting and mediate singlet exciton fission in pentacene. *Phys. Rev. Lett.* **110**, 226402 (2013).
15. Berkelbach, T. C., Hybertsen, M. S. & Reichman, D. R. Microscopic theory of singlet exciton fission. II. application to pentacene dimers and the role of superexchange. *J. Chem. Phys.* **138**, 114103 (2013).
16. Hestand, N. J. *et al.* Polarized absorption in crystalline pentacene: theory vs experiment. *J. Phys. Chem. C* **119**, 22137–22147 (2015).
